# Keep an Eye Out for Myasthenia Gravis Patients with an Eye Out

**DOI:** 10.3389/fneur.2014.00112

**Published:** 2014-07-01

**Authors:** A. Arturo Leis, Alan R. Moore

**Affiliations:** ^1^Center for Neuroscience and Neurological Recovery, Methodist Rehabilitation Center, Jackson, MS, USA; ^2^Muscle and Nerve PA, Saint Dominic Hospital, Jackson, MS, USA

**Keywords:** myasthenia gravis, ocular symptoms, eye trauma, blindness, enucleation

## Abstract

Eye trauma and blindness are common in the United States, with an incidence of over 2 million cases/year and 25 million blind adults, respectively. However, literature is surprisingly scarce on the potential confounding effect of eye trauma or blindness on the diagnosis of myasthenia gravis (MG), an autoimmune neuromuscular disease in which fluctuating ocular symptoms are the most distinguishing feature. We present the case of a 75-year-old man with eye enucleation referred for electrodiagnostic evaluation of the right upper limb after an accidental fall. Neurological examination showed proximal muscle weakness, but MG was not initially considered because the patient lacked the classic ocular symptoms of MG. The delay in diagnosis resulted in worsening of systemic MG symptoms, although in other patients it may have precipitated MG crisis or possibly death. Greater awareness that eye trauma or blindness can prevent expression of ocular symptoms in neuromuscular disorders is needed to avoid morbidity associated with an erroneous or delayed diagnosis.

## Introduction

The patient was a 75-year-old man referred by neurosurgery for evaluation of right cervical radiculopathy versus entrapment neuropathies involving the right upper limb. He had an accidental fall 1 month previously followed by pain in the right shoulder and neck. Several days later, he noticed fluctuating “pulling” in the neck that worsened after prolonged standing or sitting, with the head “dropping,” but the patient did not know if the head drooped because of muscles spasms or weakness. He could place his thumb on his chin and this maneuver helped to “pull up” the head. He also noticed weakness in the right upper limb, including inability to lift the arm above the head (“can’t hang a shirt up on a hanger”), but since the fall he also had difficulty holding the left arm overhead. He denied any numbness or tingling or lower limb symptoms. Orthopedic shoulder and spine surgery evaluations were unremarkable, including magnetic resonance imaging of the shoulder and neck. There was no history of rotator cuff or cervical spine surgery. Family history was unremarkable for nerve or muscle disorders, dystonia, or other neurological disorders. Past history was significant for hypertension, temporomandibular joint disorder, hernia repair, and left eye enucleation at age 4 following trauma. Pertinent review of systems included a 25-lb weight loss in the past 6 months while dieting. He also admitted to some dysphagia (“the food feels like it gets stuck in my throat”), weakness of mastication (“can’t chew”), and dysarthria (“I slur a little”), attributed to new dentures. He denied diabetes, thyroid disease, nutritional deficiency, hepatitis or liver disease, cancer, fever, nausea, vomiting, diarrhea, or rash. He was a non-smoker with no alcohol abuse. Medications included lisinopril, meloxicam, and aspirin. On examination, he was awake, alert, and appropriate. Speech was minimally dysarthric. Cranial nerves were normal except for left eye enucleation. Right pupil was reactive to light and accommodation with no ptosis in either eye. Face sensation was normal and muscles of facial expression were symmetrical without weakness. There was normal elevation of palate and tongue was midline without atrophy. However, a slight head drop was present and sternocleidomastoid and trapezius muscles were slightly weak (4 to 4+/5) bilaterally. There was slight atrophy or disuse wasting in right upper limb and shoulder girdle muscles with circumference mid-arm 29 cm on right and 30 cm on left. Tone was normal. Deep tendon reflexes were slightly hypoactive but symmetrical in upper limbs: 1+ biceps, brachioradialis, and triceps, and 1 to 2+ at the knees and 1+ at ankles. No Babinski signs, clonus, or other pathologic reflexes were noted. Sensation was diminished to monofilament light touch, temperature, and pin in scattered distributions of the right upper limb, not in a single dermatome, and also on the right face and chest, but this was subtle and not always reproducible. Sensation was also diminished in the median nerve distributions bilaterally. On manual muscle testing, there was weakness in right shoulder girdle muscles (most 3 to 4−/5) and in left shoulder girdle muscles (4/5). Right hip flexors were also weak at 4/5. Distal muscles were stronger bilaterally, with grip strength 67 lbs on right and 50 lbs on left. Gait was normal. Spurling’s sign was equivocal with head deviation in either direction evoking right upper shoulder pain. No scapular winging was noted. No rash, dystrophic skin or nail changes, temperature change, discoloration, or edema was present, but skin damage from chronic sun exposure was present.

**Figure 1:**
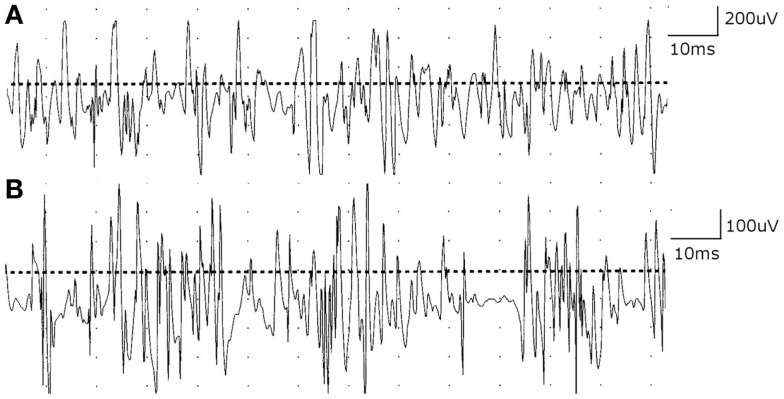
**(A)** Motor unit potentials (MUPs) in deltoid muscle are normal in phases and slightly decreased in duration and amplitude with full recruitment. **(B)** Cervical paraspinal muscles show MUPs that are short duration and low amplitude with early recruitment.

Nerve conduction studies suggested incidental bilateral median mononeuropathies at the wrists. Axillary motor responses were slightly reduced in amplitude bilaterally (right 2.6 mV; left 3.8 mV; normal ≥4.0 mV). Otherwise, nerve conduction studies in the upper limbs were unremarkable. Needle EMG examination of multiple muscles of the right upper limb, including infraspinatus and trapezius, showed no evidence of acute or chronic denervation. However, motor unit potentials (MUPs) in shoulder girdle muscles were normal to slightly increased in phases and decreased in duration and amplitude with full recruitment (Figure 1). Cervical paraspinal muscles also showed MUPs that were often short duration and low amplitude. The electrodiagnostic impression was that of a possible myopathic process affecting face, neck, and shoulders, based on the head drop and weakness in sternocleidomastoid, trapezius, and shoulder girdle muscles bilaterally, and needle EMG examination that showed short duration, low amplitude MUPs. There was *no* evidence of a diffuse neuropathic process or diffuse polyneuropathy. Repetitive stimulation studies were not performed. Blood work was unremarkable including complete blood count, sedimentation rate, comprehensive metabolic panel, serum protein electrophoresis and immune fixation electrophoresis, vitamin B12, methylmalonic acid, syphilis serology, thyroid functions, anti-nuclear antibodies, rheumatoid factor, Lyme antibody, West Nile virus test, and total creatine kinase. The patient was referred for muscle biopsy, but deferred the biopsy. One month later, he was evaluated by a second neurologist (Alan R. Moore) for weakness of neck extension (“he cannot hold his head up to eat”), jaw fatigue while chewing, difficulty swallowing solids and liquids, and a change in speech. These symptoms worsened as the day progressed. On examination, he had ptosis, bifacial weakness, dysarthria, and weakness of neck extensors and shoulder girdle muscles, but no overt external ophthalmoplegia. Repetitive stimulation studies of the median and ulnar nerves showed a post-synaptic defect in neuromuscular transmission (15 and 16% decrement in median and ulnar nerves, respectively). Although single fiber EMG in the diagnosis of myasthenia gravis (MG) is usually reserved for selected patients in whom other tests have been negative or equivocal, the lack of a characteristic history and the inability to manifest the classic ocular symptoms of MG prompted the second neurologist to proceed with single fiber EMG of the frontalis muscle, which showed increased variability of latencies among muscle fibers in a single motor unit (i.e., increased “jitter”) with blocking and a mean consecutive difference (MCD) value of 188 μs. The electrodiagnostic and clinical impression was generalized MG severely affecting bulbar and proximal upper limb muscles. A markedly elevated acetylcholine receptor binding antibody of 72.46 (normal <0.3 nmol/L) confirmed the diagnosis of MG.

## Background

Ocular symptoms are the most distinguishing feature of MG, with weakness involving extraocular muscles, eyelid levators, and orbicularis oculi leading to diplopia or ptosis (1, 2). Approximately 60–70% of patients initially present with isolated ocular symptoms (1, 3), with 50–90% of them progressing to develop weakness of bulbar and limb muscles within the first 3 years (1, 4). Eventually, about 80–85% of MG patients will develop ocular symptoms within 2 years (2, 5). In contrast, initial symptoms of oropharyngeal muscle weakness, dysarthria, dysphagia, or mastication weakness are less common (4), occurring in about 15% of patients. An initial presentation of limb weakness occurs in only 10% (3). Head drop as the presenting feature of MG is comparatively rare (6, 7). In this case, the diagnosis of MG was not initially considered because the patient lacked the hallmark ocular manifestations of MG. There was also no overt ptosis or ophthalmoplegia in the remaining eye. Surprisingly, there is very little literature and even less didactic emphasis on the potential pitfall of diagnosing MG in patients with surgical eye removal (enucleation, exenteration, and evisceration) or other eye damage that prevents expression of ocular symptoms. A PubMed search on MG revealed 14,453 papers, while MG plus eye trauma identified just 18 papers, only 10 since 1980, and none dealing with the effect of eye injury confounding the diagnosis of MG (personal communication). A PubMed search on MG plus eye evisceration, MG plus eye enucleation, and MG plus eye exenteration yielded no papers, although there was literature on MG masquerading as ocular injury (8), and the potential benefits of eye surgery to correct the ocular deficits in some cases of MG (9).

## Discussion

Eye trauma is common in the United States, with an incidence of over 2 million cases/year (10). Blindness is also common, with the National Federation of the Blind estimating that there are over 25 million blind adults (11). In spite of these numbers, the effect of eye trauma or blindness on the diagnosis of MG has not been addressed. In the current case, enucleation and the lack of overt ptosis or ophthalmoplegia in the contralateral eye confused or delayed the correct diagnosis of MG. Enucleation may also have confounded the diagnosis of subtle ophthalmoparesis in the remaining eye. This delay resulted in worsening MG symptoms, although in other MG patients it may have precipitated life-threatening weakness requiring intubation with mechanical ventilation.

The prevalence of MG in the United States is estimated by the Myasthenia Gravis Foundation of America to be 14–20/100,000 population (0.014–0.02%) or approximately 40–60 thousand cases (3). Accordingly, there are estimated 3–5 thousand MG patients with eye trauma or blindness in this country. We speculate that the number of blind patients with MG is under-diagnosed, based on personal communication with other experienced neuromuscular specialists who have not seen cases of MG confounded by eye trauma or blindness and the lack of literature emphasizing this potential pitfall. In the current case, other factors may also have contributed to the missed diagnosis, including the fact that the patient was referred for evaluation of focal rather than generalized symptoms (i.e., post-traumatic unilateral limb symptoms), so a systemic or generalized disorder was not anticipated. When a generalized condition was considered, the reduction in duration and amplitude of MUPs on needle EMG examination was misinterpreted as reflecting damage or loss of muscle fibers, resulting in the characteristic “early recruitment.” This type of recruitment is the hallmark of myopathic disorders (12). Consequently, the patient was referred for muscle biopsy. A defect in neuromuscular transmission will also produce a loss of functional muscle fibers, resulting in early recruitment (12). However, it is not commonly emphasized in the literature that neuromuscular junction disorders may present with “myopathic” recruitment. Hence, the clinical features and needle EMG examination perpetuated the erroneous diagnosis of myopathy.

## Concluding Remarks

The major factor leading to the missed diagnosis was the inability to manifest the classic ocular symptoms of MG, due to enucleation. Enucleation may also confound the diagnosis of subtle ophthalmoparesis in the remaining eye. Keeping an eye out for MG patients with an eye out will help to avoid the morbidity and possible mortality associated with erroneous diagnoses and unnecessary diagnostic procedures.

## Conflict of Interest Statement

The authors declare that the research was conducted in the absence of any commercial or financial relationships that could be construed as a potential conflict of interest.
